# Supplementary material for: Symbiont Identity Impacts the Microbiome and Volatilome of a Model Cnidarian-Dinoflagellate Symbiosis
Source: Biology (Basel). 2023 Jul 17;12(7):1014. doi: 10.3390/biology12071014 (PMC10376011; doi:10.3390/biology12071014)
Supplement: Supplementary file 1 [file biology-12-01014-s001.zip › biology-2159292-supplementary.pdf]

Supplementary material

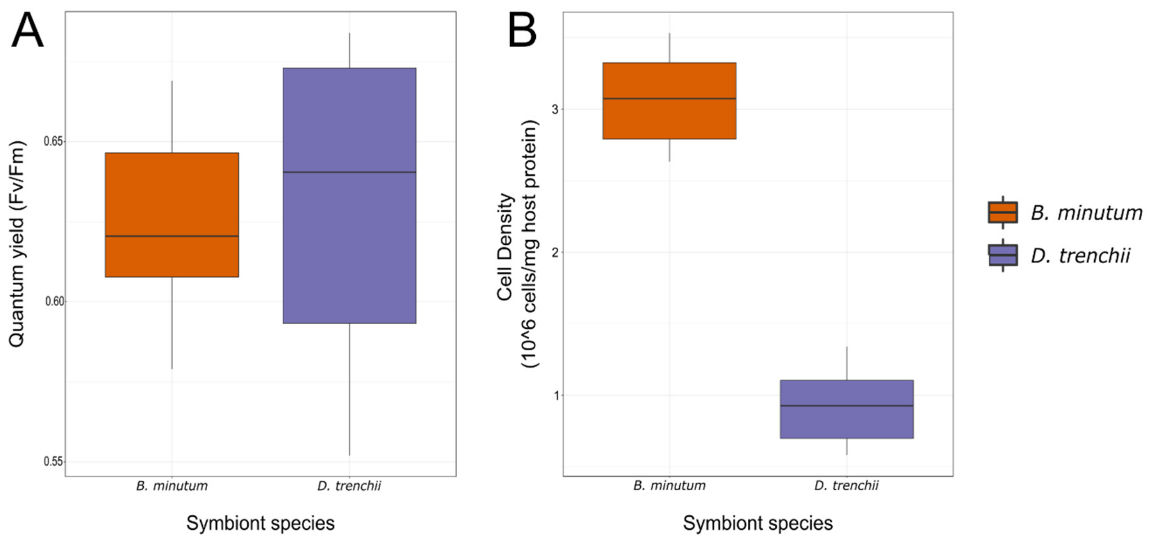

Figure S1. A) Maximum photosystem II (PSII) photochemical efficiency (F_v/F_m) and B) symbiont cell densities of holobionts populated with symbiont species *Breviolum minutum* and *Durusdinium trenchii* taken on day of BVOC and microbial sampling. Whiskers represent minimum and maximum values for this dataset, dots represent individual datapoints. Anemones were dark adapted for 15 min in sampling vials prior to quantum yield measurements.

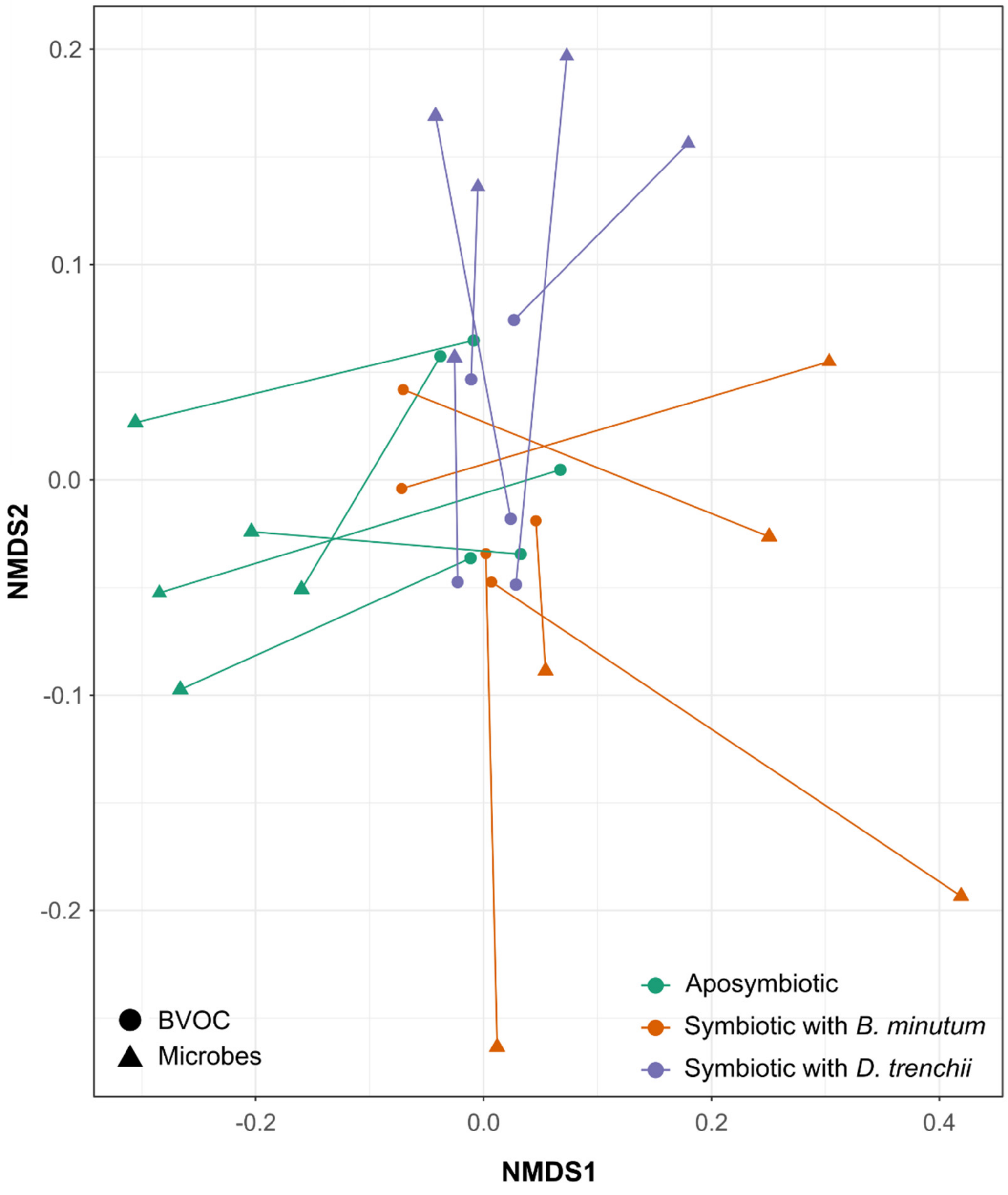

Figure S2. Correlation analysis between the microbiome and volatilome of anemones in three different symbiotic states (aposymbiosis, symbiotic with native *Breviolum minutum*, and symbiotic with non-native *Durusdinium trenchii*). Correlation was determined using Procrustes analysis on ordination distances for both datasets for all symbiotic states. This analysis calculates the shortest distance between ordination objects, in this case the microbiome and volatilome datasets; circles represent biological replicates for BVOCs, triangles represent biological replicates for microbial composition. Lines between individual circle and triangles represent distance between volatilome and microbiome community for a matched biological replicate.

Table S1. Differentially abundant BVOCs (<0.05) detected across 1) aposymbiotic anemones; 2) anemones symbiotic with *Durusdinium trenchii* (heterologous); and 3) anemones symbiotic with *B. minutum* (homologous).

BVOC	logFC	AveExpr	P.Value	adj.P.Val	label	Higher_in
1,4-Pentadiene	7.702569	8.170567	5.46E-06	0.000758	Aposymbiotic *vs. B. minutum* symbiosis	*B. minutum* symbiosis
Dimethyl sulphide	7.663887	10.45491	0.000188	0.013084	Aposymbiotic *vs. B. minutum* symbiosis	*B. minutum* symbiosis
Octanal	-6.63861	8.537077	0.001035	0.036232	Aposymbiotic *vs. B. minutum* symbiosis	Aposymbiotic anemone
Dodecanal	-6.6704	8.357859	0.001043	0.036232	Aposymbiotic *vs. B. minutum* symbiosis	Aposymbiotic anemone
Nonanal	-7.50741	8.420721	0.001484	0.041256	Aposymbiotic *vs. B. minutum* symbiosis	Aposymbiotic anemone
cis-6-Nonenol	-6.28383	7.946443	0.001877	0.043477	Aposymbiotic *vs. B. minutum* symbiosis	Aposymbiotic anemone
Dimethyl sulphide	9.898329	10.45491	5.14E-07	7.14E-05	Aposymbiotic *vs. D. trenchii* symbiosis	*D. trenchii* symbiosis
1,1,2,2,3,3-Hexamethylindane	-7.68899	6.151933	0.000151	0.010528	Aposymbiotic *vs. D. trenchii* symbiosis	Aposymbiotic anemone
2-Methoxy-thiazole	6.259711	6.76794	0.000472	0.021864	Aposymbiotic *vs. D. trenchii* symbiosis	*D. trenchii* symbiosis
1,4-Pentadiene	-7.43341	8.170567	8.48E-05	0.011789	*B. minutum symbiosis* symbiosis *vs. D. trenchii* symbiosis	*B. minutum symbiosis* symbiosis

Table S2. Differentially abundant microbes ($p < 0.05$) detected across 1) aposymbiotic anemones; 2) anemones symbiotic with *D. trenchii* (heterologous); and 3) anemones symbiotic with *B. minutum symbiosis* (homologous).

Name	taxon_ID	logFC	adj.P.Val	label	Higher in
**Campylobacteraceae**	taxon_2	-11.6377561	1.05E-09	Aposymbiotic *vs.* *B. minutum* symbiosis	Aposymbiotic anemone
**Vibrionaceae**	taxon_20	-9.083924682	3.50E-07	Aposymbiotic *vs.* *B. minutum* symbiosis	Aposymbiotic anemone
**Bacteroidetes**	taxon_35	10.98921978	3.50E-07	Aposymbiotic *vs.* *B. minutum* symbiosis	*B. minutum* symbiosis
Tepidibacter mesophilus	taxon_33	-8.019097041	4.22E-07	Aposymbiotic *vs.* *B. minutum* symbiosis	Aposymbiotic anemone
**Gammaproteobacteria**	taxon_24	-8.635219055	1.10E-06	Aposymbiotic *vs.* *B. minutum* symbiosis	Aposymbiotic anemone
**Alphaproteobacteria**	taxon_15	-9.14649014	1.10E-06	Aposymbiotic *vs.* *B. minutum* symbiosis	Aposymbiotic anemone
**Paraglaciecola**	taxon_63	8.054035612	6.72E-06	Aposymbiotic *vs.* *B. minutum* symbiosis	*B. minutum* symbiosis
**Proteobacteria**	taxon_155	6.774446978	1.02E-05	Aposymbiotic *vs.* *B. minutum* symbiosis	*B. minutum* symbiosis
**Vibrionaceae**	taxon_55	-5.933931872	3.38E-05	Aposymbiotic *vs.* *B. minutum* symbiosis	Aposymbiotic anemone
**Flavobacteriia**	taxon_30	-6.89165268	4.99E-05	Aposymbiotic *vs.* *B. minutum* symbiosis	Aposymbiotic anemone
**Alteromonadaceae**	taxon_131	6.698017115	8.15E-05	Aposymbiotic *vs.* *B. minutum* symbiosis	*B. minutum* symbiosis
**unclassified**	taxon_67	-6.00368853	9.73E-05	Aposymbiotic *vs.* *B. minutum* symbiosis	Aposymbiotic anemone
**Vibrionaceae**	taxon_19	-6.881473481	0.00012738	Aposymbiotic *vs.* *B. minutum* symbiosis	Aposymbiotic anemone
**Bacteroidetes**	taxon_8	-8.38106729	0.00012738	Aposymbiotic *vs.* *B. minutum* symbiosis	Aposymbiotic anemone
**Rhodobacteraceae**	taxon_117	6.344366813	0.000258354	Aposymbiotic *vs.* *B. minutum* symbiosis	*B. minutum* symbiosis
Polaribacter huanghezhanensis	taxon_62	-5.346774006	0.000258354	Aposymbiotic *vs.* *B. minutum* symbiosis	Aposymbiotic anemone

Vibrio sinaloensis	taxon_43	-5.633854016	0.000258354	Aposymbiotic *vs.* *B. minutum* symbiosis	Aposymbiotic anemone
Vibrio crosai	taxon_42	-5.38260227	0.00034632	Aposymbiotic *vs.* *B. minutum* symbiosis	Aposymbiotic anemone
**Bacteroidetes**	taxon_29	-6.516638661	0.00034632	Aposymbiotic *vs.* *B. minutum* symbiosis	Aposymbiotic anemone
**Alteromonadaceae**	taxon_95	6.143878047	0.000749625	Aposymbiotic *vs.* *B. minutum* symbiosis	*B. minutum* symbiosis
***Chlamydiia* sp.**	taxon_79	6.594860407	0.000756115	Aposymbiotic *vs.* *B. minutum* symbiosis	*B. minutum* symbiosis
**Rhodospirillales**	taxon_187	5.115962477	0.001283903	Aposymbiotic *vs.* *B. minutum* symbiosis	*B. minutum* symbiosis
**Flavobacteriales**	taxon_146	5.28403114	0.001283903	Aposymbiotic *vs.* *B. minutum* symbiosis	*B. minutum* symbiosis
***Labrenzia* sp.**	taxon_161	4.929289156	0.001283903	Aposymbiotic *vs.* *B. minutum* symbiosis	*B. minutum* symbiosis
***Chlamydiia* sp.**	taxon_176	4.912479847	0.001283903	Aposymbiotic *vs.* *B. minutum* symbiosis	*B. minutum* symbiosis
Vibrio crosai	taxon_39	-5.322736099	0.001283903	Aposymbiotic *vs.* *B. minutum* symbiosis	Aposymbiotic anemone
**Gammaproteobacteria**	taxon_143	4.854329288	0.001444656	Aposymbiotic *vs.* *B. minutum* symbiosis	*B. minutum* symbiosis
***Parasphingorhabdus* sp.**	taxon_169	4.72459138	0.002352881	Aposymbiotic *vs.* *B. minutum* symbiosis	*B. minutum* symbiosis
Maritalea porphyrae	taxon_75	-4.857059267	0.002448795	Aposymbiotic *vs.* *B. minutum* symbiosis	Aposymbiotic anemone
***Cohaesibacter* sp.**	taxon_50	-4.993792627	0.002596865	Aposymbiotic *vs.* *B. minutum* symbiosis	Aposymbiotic anemone
**Rhodobacteraceae**	taxon_17	-4.838214878	0.0029103	Aposymbiotic *vs.* *B. minutum* symbiosis	Aposymbiotic anemone
Vibrio crosai	taxon_11	-3.901426652	0.006335499	Aposymbiotic *vs.* *B. minutum* symbiosis	Aposymbiotic anemone
Pseudoalteromonas piscicida	taxon_93	-4.11271875	0.006335499	Aposymbiotic *vs.* *B. minutum* symbiosis	Aposymbiotic anemone
***Arcomobacter* sp.**	taxon_18	-5.82323865	0.008380197	Aposymbiotic *vs.* *B. minutum* symbiosis	Aposymbiotic anemone

**Flavobacteriaceae**	taxon_4	-6.858945282	0.009745443	Aposymbiotic *vs.* *B. minutum* symbiosis	Aposymbiotic anemone
***Croceibacter atlanticus* HTCC2559**	taxon_68	-3.715856291	0.012102204	Aposymbiotic *vs.* *B. minutum* symbiosis	Aposymbiotic anemone
**unclassified**	taxon_38	6.385172422	0.013491098	Aposymbiotic *vs.* *B. minutum* symbiosis	*B. minutum* symbiosis
Vibrio sinaloensis	taxon_44	-3.926324676	0.017519447	Aposymbiotic *vs.* *B. minutum* symbiosis	Aposymbiotic anemone
**Proteobacteria**	taxon_166	2.993939404	0.022098695	Aposymbiotic *vs.* *B. minutum* symbiosis	*B. minutum* symbiosis
**Rhodobacteraceae**	taxon_45	-4.044200152	0.032038917	Aposymbiotic *vs.* *B. minutum* symbiosis	Aposymbiotic anemone
**Flavobacteriaceae**	taxon_78	3.906151746	0.047384012	Aposymbiotic *vs.* *B. minutum* symbiosis	*B. minutum* symbiosis
**Campylobacteraceae**	taxon_2	-12.54558197	1.37E-10	Aposymbiotic *vs.* *D. trenchii* symbiosis	Aposymbiotic anemone
Vibrio crosai	taxon_42	-8.720940695	2.00E-07	Aposymbiotic *vs.* *D. trenchii* symbiosis	Aposymbiotic anemone
**Gammaproteobacteria**	taxon_24	-9.543044922	2.29E-07	Aposymbiotic *vs.* *D. trenchii* symbiosis	Aposymbiotic anemone
**Vibrionaceae**	taxon_55	-7.697699825	4.13E-07	Aposymbiotic *vs.* *D. trenchii* symbiosis	Aposymbiotic anemone
Tepidibacter mesophilus	taxon_33	-7.925390564	8.02E-07	Aposymbiotic *vs.* *D. trenchii* symbiosis	Aposymbiotic anemone
Vibrio sinaloensis	taxon_43	-7.632038079	4.15E-06	Aposymbiotic *vs.* *D. trenchii* symbiosis	Aposymbiotic anemone
**Gammaproteobacteria**	taxon_61	-7.094961195	9.14E-06	Aposymbiotic *vs.* *D. trenchii* symbiosis	Aposymbiotic anemone
Vibrio sinaloensis	taxon_44	-7.397723174	2.18E-05	Aposymbiotic *vs.* *D. trenchii* symbiosis	Aposymbiotic anemone
**Bacteroidetes**	taxon_8	-9.288893157	3.38E-05	Aposymbiotic *vs.* *D. trenchii* symbiosis	Aposymbiotic anemone
Polaribacter huanghezhanensis	taxon_62	-6.254599873	3.81E-05	Aposymbiotic *vs.* *D. trenchii* symbiosis	Aposymbiotic anemone
**unclassified**	taxon_67	-6.352450017	4.50E-05	Aposymbiotic *vs.* *D. trenchii* symbiosis	Aposymbiotic anemone

**Flavobacteriia**	taxon_30	-6.995921603	4.50E-05	Aposymbiotic *vs.* *D. trenchii* symbiosis	Aposymbiotic anemone
Cohaesibacter	taxon_50	-7.07681947	4.50E-05	Aposymbiotic *vs.* *D. trenchii* symbiosis	Aposymbiotic anemone
**Bacteroidetes**	taxon_29	-7.424464528	6.46E-05	Aposymbiotic *vs.* *D. trenchii* symbiosis	Aposymbiotic anemone
Croceibacter atlanticus **HTCC2559**	taxon_68	-5.927742528	9.47E-05	Aposymbiotic *vs.* *D. trenchii* symbiosis	Aposymbiotic anemone
Vibrio crosai	taxon_39	-6.412746239	0.000242384	Aposymbiotic *vs.* *D. trenchii* symbiosis	Aposymbiotic anemone
**Alphaproteobacteria**	taxon_15	-6.581707156	0.000333199	Aposymbiotic *vs.* *D. trenchii* symbiosis	Aposymbiotic anemone
**Proteobacteria**	taxon_166	5.341653404	0.000428386	Aposymbiotic *vs.* *D. trenchii* symbiosis	*D. trenchii* symbiosis
**Gammaproteobacteria**	taxon_128	5.615738594	0.000664363	Aposymbiotic *vs.* *D. trenchii* symbiosis	*D. trenchii* symbiosis
Pseudoalteromonas piscicida	taxon_93	-5.020544617	0.001218664	Aposymbiotic *vs.* *D. trenchii* symbiosis	Aposymbiotic anemone
Vibrio crosai	taxon_11	-4.583049866	0.001537553	Aposymbiotic *vs.* *D. trenchii* symbiosis	Aposymbiotic anemone
**Rhizobiales**	taxon_65	5.977279081	0.002575151	Aposymbiotic *vs.* *D. trenchii* symbiosis	*D. trenchii* symbiosis
Arcobacter	taxon_18	-6.731064517	0.002718679	Aposymbiotic *vs.* *D. trenchii* symbiosis	Aposymbiotic anemone
Erythrobacter gaetbuli	taxon_175	4.912908552	0.002765546	Aposymbiotic *vs.* *D. trenchii* symbiosis	*D. trenchii* symbiosis
**Myxococcales**	taxon_66	5.415370989	0.007021348	Aposymbiotic *vs.* *D. trenchii* symbiosis	*D. trenchii* symbiosis
**Cytophagales**	taxon_121	4.396457203	0.013454925	Aposymbiotic *vs.* *D. trenchii* symbiosis	*D. trenchii* symbiosis
**unclassified**	taxon_119	4.634983407	0.013531188	Aposymbiotic *vs.* *D. trenchii* symbiosis	*D. trenchii* symbiosis
**Flavobacteriaceae**	taxon_122	-3.856422311	0.017260377	Aposymbiotic *vs.* *D. trenchii* symbiosis	Aposymbiotic anemone
**Gammaproteobacteria**	taxon_174	4.113334599	0.017260377	Aposymbiotic *vs.* *D. trenchii* symbiosis	*D. trenchii* symbiosis

**Bacteroidetes**	taxon_54	-4.217438934	0.017368823	Aposymbiotic *vs.* *D. trenchii* symbiosis	Aposymbiotic anemone
***Photobacterium gaetbulicola* Gung47**	taxon_52	-4.217631671	0.018057609	Aposymbiotic *vs.* *D. trenchii* symbiosis	Aposymbiotic anemone
**Oceanospirillaceae**	taxon_120	-4.06160099	0.018057609	Aposymbiotic *vs.* *D. trenchii* symbiosis	Aposymbiotic anemone
**Vibrionaceae**	taxon_20	-3.385733945	0.020879077	Aposymbiotic *vs.* *D. trenchii* symbiosis	Aposymbiotic anemone
***Limimaricola cinnabarinus* LL-001**	taxon_240	3.849855431	0.020879077	Aposymbiotic *vs.* *D. trenchii* symbiosis	*D. trenchii* symbiosis
**Rhodobacteraceae**	taxon_241	3.462514472	0.022291686	Aposymbiotic *vs.* *D. trenchii* symbiosis	*D. trenchii* symbiosis
***Limimaricola* sp.**	taxon_84	3.703038546	0.026208693	Aposymbiotic *vs.* *D. trenchii* symbiosis	*D. trenchii* symbiosis
**Vibrionaceae**	taxon_19	-3.528020008	0.027453462	Aposymbiotic *vs.* *D. trenchii* symbiosis	Aposymbiotic anemone
**Alphaproteobacteria**	taxon_106	-4.091487448	0.030099188	Aposymbiotic *vs.* *D. trenchii* symbiosis	Aposymbiotic anemone
**Bacteroidetes**	taxon_35	-8.313224035	0.000316696	*B. minutum* symbiosis *vs. D. trenchii* symbiosis	*B. minutum* symbiosis
**Paraglaciecola**	taxon_63	-6.907999642	0.000316696	*B. minutum* symbiosis *vs. D. trenchii* symbiosis	*B. minutum* symbiosis
**Proteobacteria**	taxon_155	-5.742388621	0.000326894	*B. minutum* symbiosis *vs. D. trenchii* symbiosis	*B. minutum* symbiosis
**Rhodobacteraceae**	taxon_45	7.045217916	0.000570146	*B. minutum* symbiosis *vs. D. trenchii* symbiosis	*D. trenchii* symbiosis
**Alteromonadaceae**	taxon_95	-6.389639085	0.00116332	*B. minutum* symbiosis *vs. D. trenchii* symbiosis	*B. minutum* symbiosis
**Alteromonadaceae**	taxon_131	-5.665958758	0.001373034	*B. minutum* symbiosis *vs. D. trenchii* symbiosis	*B. minutum* symbiosis
**Vibrionaceae**	taxon_20	5.698190737	0.001862178	*B. minutum* symbiosis *vs. D.*	*D. trenchii* symbiosis

				trenchii symbiosis	
**Rhodobacteraceae**	taxon_117	-5.312308456	0.004100782	*B. minutum* symbiosis *vs. D. trenchii* symbiosis	*B. minutum* symbiosis
***Chlamydiia* sp.**	taxon_79	-5.56280205	0.010863159	*B. minutum* symbiosis *vs. D. trenchii* symbiosis	*B. minutum* symbiosis
**Gammaproteobacteria**	taxon_61	-4.225456091	0.022884636	*B. minutum* symbiosis *vs. D. trenchii* symbiosis	*B. minutum* symbiosis
**Rhodospirillales**	taxon_187	-4.08390412	0.022884636	*B. minutum* symbiosis *vs. D. trenchii* symbiosis	*B. minutum* symbiosis
**Rhodobacteraceae**	taxon_17	4.325985709	0.022884636	*B. minutum* symbiosis *vs. D. trenchii* symbiosis	*D. trenchii* symbiosis
***Labrenzia* sp.**	taxon_161	-3.897230799	0.022884636	*B. minutum* symbiosis *vs. D. trenchii* symbiosis	*B. minutum* symbiosis
***Chlamydiia* sp.**	taxon_176	-3.88042149	0.022884636	*B. minutum* symbiosis *vs. D. trenchii* symbiosis	*B. minutum* symbiosis

Table S3. BVOCs detected throughout dataset. All BVOCs (peak normalised to protein content) and their chemical classes that were detected in aposymbiotic anemones, anemones symbiotic with *B. minutum symbiosis* (homologous symbiosis) and anemones symbiotic with *D. trenchii* (heterologous symbiosis). Compounds had to be detected in at least four replicates in at least one symbiotic state. Chemical class was determined based on the molecule's functional group(s). Significance was determined using differential abundance testing and the number of asterisks denotes the size of the adjusted p-value: *<0.05, **<0.01, ***<0.001.

Colour scale *		
Zero	Medium	High

BVOC	Homologous symbiosis								Heterologous symbiosis								Aposymbiotic anemone								Functional group	Significance
(2,4-cyclopentadien-1-ylidenemethyl)-benzene	4.E+02	0	1.E+03	0	1.E+03	0	2.E+03	0	0	0	4.E+03	0	5.E+03	0	5.E+03	0	0	3.E+02	4.E+03	0	0	0	4.E+03	0	Aromatic compound	
(2-methyl-n-3-propenyl)-(pentamethylcyclopentadienyl)-cobalt	0	3.E+03	0	4.E+03	0	0	0	0	0	2.E+04	0	0	0	1.E+04	0	0	3.E+04	0	0	1.E+04	0	3.E+02	0	3.E+03	Cobalt containing compound	
(E)-Hex-3-enyl (E)-2-methylbut-2-enoate	1.E+02	1.E+02	0	0	2.E+02	0	2.E+02	0	0	0	0	1.E+03	0	0	0	0	0	0	9.E+02	0	0	0	0	0	Ester	
1-(1-propynyl)-cyclohexene	0	2.E+01	0	5.E+01	0	0	0	0	2.E+02	3.E+02	4.E+02	2.E+02	0	1.E+02	2.E+02	1.E+01	0	0	4.E+02	3.E+02	2.E+02	2.E+02	2.E+02	1.E+02	Alkyne	
1-(6-methoxy-2-naphthyl)-ethanol	4.E+01	0	8.E+01	0	5.E+01	0	4.E+01	0	9.E+01	0	9.E+01	0	2.E+02	0	9.E+01	0	0	2.E+02	3.E+02	0	2.E+02	0	8.E+01	0	DFG	
1,1,2,2,3,3-Hexamethylindane	1.E+02	0	0	0	0	0	8.E+01	0	0	0	0	0	0	0	0	0	0	4.E+02	0	1.E+02	3.E+02	1.E+02	3.E+02	5.E+01	Aromatic compound	*
1,1,3-Trimethylindane	2.E+02	0	0	0	0	0	0	0	4.E+02	0	0	0	3.E+02	0	2.E+02	0	0	5.E+02	8.E+02	0	4.E+02	0	3.E+02	0	Aromatic compound	
1,1-dimethyl-1H-indene	3.E+01	0	0	0	0	0	5.E+01	0	0	2.E+02	1.E+02	0	1.E+01	0	9.E+01	0	0	1.E+02	2.E+02	0	4.E+01	0	8.E+01	0	Aromatic compound	
1,2,4-Oxadiazole-3-Methyl-5-pyrid-2-yl	3.E+01	6.E+01	0	0	7.E+01	0	5.E+01	0	0	2.E+02	1.E+02	0	1.E+02	2.E+02	0	0	0	0	2.E+02	6.E+01	0	0	2.E+01	0	DFG	
1,3-Pentanediol, 2,2,4-trimethyl, 1-isobutyrate	0	0	0	0	2.E+01	0	2.E+02	0	1.E+01	2.E+03	2.E+02	0	0	3.E+02	2.E+02	0	3.E+02	3.E+02	0	6.E+02	0	0	0	0	DFG	
1,4-Pentadiene	2.E+02	2.E+02	3.E+02	3.E+02	5.E+02	4.E+02	8.E+02	8.E+02	0	0	0	0	1.E+02	0	3.E+02	3.E+02	0	4.E+02	0	0	0	0	0	0	Alkene	***
10,13-dimethyl-, methyl ester tetradecanoic acid	0	0	7.E+01	0	9.E+01	0	1.E+02	0	0	4.E+02	0	4.E+02	0	0	3.E+02	0	8.E+02	0	3.E+02	0	0	4.E+02	0	4.E+02	Ester	
1-Dimethylamino-2-propyne	0	4.E+02	1.E+02	0	2.E+02	0	2.E+02	0	4.E+02	0	0	0	8.E+02	0	0	0	0	0	2.E+03	0	4.E+02	0	1.E+03	0	Nitrogen containing compound	
1-Methoxy-1-buten-3-yne	2.E+01	0	2.E+01	0	3.E+00	0	6.E+00	0	8.E+01	8.E+01	2.E+02	1.E+01	1.E-01	0	8.E+00	0	1.E+02	2.E+02	2.E+02	0	0	0	0	0	Ether	
1-methyl-1H-Pyrrole	7.E+02	2.E+03	5.E+02	7.E+02	2.E+03	2.E+03	2.E+03	1.E+03	1.E+03	4.E+02	7.E+03	2.E+03	5.E+03	8.E+03	4.E+03	2.E+03	9.E+02	4.E+02	7.E+02	1.E+03	2.E+03	1.E+03	1.E+03	2.E+03	Nitrogen containing compound	
1-Methylethyl-benzene	7.E+02	0	5.E+02	0	2.E+02	0	2.E+02	0	1.E+03	0	1.E+03	0	4.E+02	0	4.E+02	0	3.E+02	3.E+03	4.E+03	0	8.E+02	0	5.E+02	0	Aromatic compound	
1-Methylnapthalene	8.E+02	0	1.E+03	0	1.E+03	0	2.E+03	0	9.E+02	0	8.E+03	0	5.E+03	0	4.E+03	0	0	4.E+03	1.E+04	0	6.E+03	0	4.E+03	0	Aromatic compound	
1-Nitroadamantane	0	0	0	0	0	0	1.E+02	0	0	2.E+02	2.E+02	4.E+02	0	2.E+02	0	0	1.E+02	0	6.E+02	0	2.E+02	7.E+00	0	0	DFG	
2-(1-methylpropyl)-phenol	9.E+02	0	3.E+03	0	0	5.E+01	0	0	0	2.E+03	0	0	0	9.E+02	3.E+03	0	3.E+03	4.E+02	1.E+03	2.E+03	1.E+03	0	0	2.E+03	Alcohol	
2-(4-Formyl-phenoxy)-acetamide	5.E+01	7.E+01	1.E+01	0	6.E-01	0	5.E+02	5.E+01	0	1.E+03	3.E+02	3.E+02	5.E+02	2.E+02	9.E+01	0	1.E+03	2.E+01	2.E+03	1.E+02	4.E+02	2.E+02	0	0	DFG	
2,2-dimethyl-, ethenyl ester pentanoic acid	0	0	7.E+01	1.E+02	6.E+01	0	0	0	6.E+01	0	0	9.E+02	0	4.E+02	0	7.E+00	3.E+02	0	1.E+03	5.E+02	2.E+02	3.E+02	4.E+02	0	Ester	
2,3,3-trimethylcyclobutanone	0	2.E+00	4.E+01	5.E+01	0	0	0	0	2.E+02	0	2.E+02	4.E+02	0	0	0	0	1.E+02	1.E+02	6.E+02	1.E+02	2.E+02	0	0	0	Ketone	
2,3-Butanedione	0	2.E+02	3.E+02	6.E+01	2.E+02	0	0	0	1.E+03	0	1.E+03	0	0	0	0	0	7.E+01	0	3.E+03	0	2.E+02	2.E+02	0	0	Ketone	
2,3-Dihydro-4-methoxyindole-2-one	2.E+02	2.E+02	2.E+02	1.E+02	9.E+01	1.E+02	3.E+02	2.E+02	0	5.E+02	5.E+02	0	4.E+02	6.E+02	3.E+02	8.E+01	0	3.E+02	8.E+02	6.E+02	6.E+02	7.E+02	3.E+02	2.E+02	DFG	
2,5,9-Trimethyldecane	2.E+02	0	0	0	0	4.E+02	6.E+02	1.E+02	0	2.E+03	2.E+03	2.E+03	2.E+03	2.E+03	2.E+03	1.E+02	2.E+03	0	4.E+03	1.E+03	2.E+03	2.E+03	1.E+03	4.E+02	Alkane	
2,6-bis(1,1-dimethylethyl)-4-ethylphenol	0	0	5.E+01	0	2.E+01	4.E+01	1.E+02	3.E+01	0	5.E+02	2.E+02	1.E+02	0	0	2.E+02	0	3.E+02	0	5.E+02	4.E+01	0	0	0	0	Alcohol	
2-Benzylaminoacetonitrile	8.E+00	0	0	0	2.E+02	8.E+01	2.E+02	0	0	3.E+02	8.E+02	8.E+01	6.E+02	2.E+02	5.E+02	2.E+02	2.E+02	0	6.E+02	1.E+02	6.E+02	5.E+02	3.E+02	0	Nitrogen containing compound	
2-bromopropane	0	0	1.E+02	1.E+01	0	0	0	5.E+02	0	0	0	5.E+01	0	0	0	0	0	0	8.E+03	0	3.E+02	8.E+02	2.E+01	0	Halogenated hydrocarbon	
2-Chloro-2,2-difluoro-1-phenylethanone	0	0	0	0	2.E+01	0	3.E+01	3.E+01	0	4.E+02	3.E+02	1.E+03	0	0	2.E+02	0	7.E+02	0	2.E+02	1.E+02	1.E+02	3.E+02	0	0	DFG	
2-Ethyl-1-hexanol	0	0	1.E+03	0	9.E+02	0	0	0	7.E+03	4.E+03	1.E+02	0	0	3.E+02	0	0	1.E+03	1.E+03	3.E+03	0	0	0	0	0	Alcohol	
2-Ethylhexyl hexyl ester sulfurous acid	3.E+02	0	2.E+02	0	7.E+01	1.E+01	4.E+02	0	0	9.E+02	5.E+02	0	8.E+02	0	8.E+02	0	2.E+03	2.E+03	3.E+03	4.E+02	1.E+03	2.E+02	1.E+03	0	Sulphur containing compound	
2-Hydroxyoctyl pentyl sulfoxide	0	0	7.E+01	0	0	5.E+01	0	4.E+01	1.E+02	0	6.E+01	0	2.E+02	0	7.E+01	0	9.E+01	0	6.E+02	1.E+02	1.E+02	1.E+02	0	1.E+02	DFG	
2-Methoxyresorcinol	2.E+02	4.E+02	0	5.E+02	0	3.E+02	0	2.E+02	0	6.E+02	8.E+02	9.E+02	7.E+02	7.E+02	7.E+02	3.E+02	8.E+02	5.E+02	2.E+03	1.E+03	0	1.E+03	0	9.E+02	DFG	
2-Methoxy-thiazole	0	8.E+01	0	0	0	0	4.E+02	5.E+01	0	3.E+02	2.E+02	0	6.E+02	8.E+02	2.E+02	2.E+02	0	0	0	0	0	0	0	0	DFG	*
2-Methyl-1,3,6-Trioxocane	6.E+01	6.E+01	5.E+00	2.E+01	0	0	7.E+01	3.E+01	3.E+01	0	0	0	8.E+01	2.E+02	8.E+00	0	6.E+01	8.E+01	2.E+02	2.E+02	2.E+02	1.E+02	0	0	Ether	
2-Methyl-1-propene	6.E+01	0	0	0	0	0	0	0	2.E+02	7.E+02	0	3.E+02	0	0	0	1.E+02	3.E+02	0	0	0	0	0	0	1.E+02	Alkene	
2-Methyl-2-phenyl-1,3-benzodioxole	4.E+01	0	1.E+02	0	6.E+01	0	2.E+01	0	2.E+02	6.E+02	8.E+01	4.E+01	0	2.E+01	1.E+02	0	1.E+02	6.E+01	2.E+02	0	6.E+01	2.E+03	0	0	Ether	

Table S3 (cont.).

**2-Methyl-2-propanol**	0	0	0	2.E+02	0	0	3.E+02	0	0	0	3.E+02	0	0	0	4.E+01	0	0	0	3.E+03	6.E+02	2.E+02	2.E+03	0	4.E+02	Alcohol	
**2-Methylpropyl ester butanoic acid**	0	0	0	0	0	0	0	0	1.E+01	0	0	0	0	0	0	0	9.E+02	7.E+02	7.E+02	7.E+02	0	3.E+02	0	0	Ester	
**2-Propenal**	0	0	0	0	0	0	0	0	5.E+02	4.E+02	0	5.E+02	0	0	6.E+02	0	0	0	1.E+03	0	0	0	0	0	Aldehyde	
**3-(3,4-dimethoxyphenyl), (E)-2-propenoic acid**	0	1.E+02	2.E+02	0	5.E+01	6.E+00	0	0	0	3.E+02	0	0	3.E+02	2.E+02	3.E+01	0	3.E+02	0	0	0	6.E+02	8.E+02	0	2.E+01	DFG	
**3-(4-Methoxyphenyl)-3-methyl-2,5-pyrrolidinedione**	0	0	0	0	0	0	4.E+01	6.E+01	0	1.E+02	0	0	0	1.E+02	0	0	0	0	2.E+02	2.E+02	0	2.E+02	9.E+01	0	DFG	
**3,3,4-Trimethylheptane**	0	6.E+01	0	4.E+02	4.E+02	0	2.E+02	0	9.E+02	1.E+02	0	0	3.E+02	1.E+03	0	0	4.E+02	0	7.E+02	2.E+03	2.E+01	0	0	0	Alkane	
**3,3,5-Trimethylcyclohexyl methacrylate**	0	0	3.E+03	0	3.E+03	0	4.E+03	0	1.E+03	0	9.E+03	0	1.E+04	0	1.E+04	0	0	0	2.E+04	0	1.E+04	0	1.E+04	0	Ester	
**3,3-Dimethylpentane**	0	0	0	0	0	0	2.E+02	0	3.E+02	0	0	6.E+02	0	0	8.E+02	3.E+02	8.E+02	0	1.E+03	9.E+02	0	0	7.E+02	0	Alkane	
**3,4-Methylenedioxy-N-ethylamphetamine**	0	1.E+02	1.E+02	0	0	0	0	0	0	4.E+02	2.E+02	3.E+02	0	0	0	0	2.E+02	0	6.E+02	0	0	0	2.E+02	1.E+02	DFG	
**3-Chloro-1H-1,2,4-triazole**	0	0	3.E+01	0	0	0	6.E+01	0	0	5.E+02	3.E+02	0	8.E+01	0	0	0	7.E+01	1.E+02	2.E+02	0	2.E+01	2.E+01	0	5.E+01	DFG	
**3-Methyl-3-phenyl-azetidine**	5.E+01	0	5.E+01	0	4.E-01	0	9.E-01	0	1.E+02	0	1.E+02	0	0	0	0	0	0	4.E+02	3.E+02	0	3.E+01	0	6.E+00	0	Nitrogen containing compound	
**3-Methylcyclopentyl acetate**	0	1.E+02	0	0	0	0	0	0	0	0	5.E+01	3.E+02	0	0	0	0	1.E+02	0	0	3.E+02	0	1.E+02	1.E+01	0	Ester	
**3-Methylnonane**	0	0	0	0	8.E+01	0	2.E+02	0	0	0	8.E+02	0	0	0	4.E+02	2.E+02	1.E+03	1.E+03	2.E+03	0	0	0	6.E+02	0	Alkane	
**3-Octadecyne**	0	2.E+01	4.E+01	0	8.E+01	0	1.E+02	0	0	4.E+02	4.E+02	3.E+02	1.E+02	0	2.E+02	0	5.E+02	0	1.E+03	2.E+02	0	4.E+02	3.E+02	1.E+02	Alkyne	
**4-(1,1-dimethylethyl)-benzenepropanal**	0	0	0	0	3.E+01	0	2.E+01	0	0	4.E+02	0	3.E+02	0	4.E+02	0	0	8.E+02	0	0	7.E+02	0	5.E+02	0	4.E+02	Aldehyde	
**4-(4-butylcyclohexyl)-2,3-dicyano-4-ethoxyphenyl ester benzoic acid**	1.E+01	2.E+01	0	0.E+00	1.E+01	0	1.E+01	0	0	0	0	0	0	0	0	0	1.E+02	0	0	0	0	0	0	0	DFG	
**4-Ethoxybenzaldehyde**	4.E+02	5.E+02	0	4.E+02	0	0	5.E+02	3.E+02	0	0	3.E+02	0	8.E+02	0	8.E+02	8.E+02	2.E+03	0	5.E+02	1.E+03	1.E+03	1.E+03	0	2.E+02	DFG	
**4-Hydroxy-2-methylacetophenone**	1.E+02	0	0	2.E+01	5.E+01	0	5.E+01	3.E+01	2.E+03	1.E+03	0	1.E+01	3.E+02	1.E+02	6.E+02	2.E+01	7.E+02	1.E+03	0	5.E+02	0	7.E+02	9.E+01	2.E+02	DFG	
**5-Methyl-3-isoxazolamine**	9.E+01	2.E+02	1.E+02	2.E+02	0	0	0	0	3.E+02	4.E+02	0	4.E+02	0	0	0	0	0	4.E+02	9.E+02	5.E+02	4.E+02	5.E+02	4.E+02	4.E+02	DFG	
**6,10-Dimethyl-5,9-undecadien-2-one**	0	0	0	0	0	0	0	0	0	0	8.E+02	0	0	0	6.E+02	0	1.E+02	2.E+02	1.E+03	0	2.E+02	5.E+02	0	0	Ketone	
**a-amino-, methyl ester benzeneacetic acid**	0	2.E+01	0	7.E+00	0	0	7.E+01	4.E+01	0	1.E+02	1.E+02	2.E+02	0	6.E+01	6.E+01	9.E+01	4.E+02	0	6.E+01	0	3.E+02	2.E+02	0	0	DFG	
**Acetaldehyde**	0	0	2.E+03	0	0	0	0	0	0	0	0	0	0	0	9.E+03	0	0	0	2.E+04	0	2.E+03	7.E+03	7.E+03	0	Aldehyde	
**Acetone**	0	2.E+02	2.E+03	2.E+03	0	0	4.E+01	0	2.E+03	0	2.E+03	0	0	0	0	0	0	0	3.E+04	0	0	0	0	0	Ketone	
**Acetophenone**	7.E+01	0	3.E+01	0	4.E+01	0	6.E+01	0	0	1.E+03	9.E+02	2.E+02	0	0	1.E+02	0	2.E+02	6.E+02	1.E+03	1.E+01	0	0	0	0	Ketone	
**aR-Himachalene**	2.E+02	0	2.E+02	0	1.E+02	0	1.E+02	0	5.E+02	0	5.E+02	0	4.E+02	0	2.E+02	0	0	5.E+02	1.E+03	0	5.E+02	0	2.E+02	0	Aromatic compound	
**Benzaldehyde**	8.E+01	2.E+02	6.E+01	0	5.E+01	0	2.E+01	0	2.E+02	1.E+03	1.E+03	2.E+02	0	0	0	0	1.E+02	1.E+03	2.E+03	3.E+02	3.E+01	0	0	0	Aldehyde	
**Benzene**	8.E+01	0	3.E+02	0	3.E+02	0	0	0	6.E+02	6.E+02	5.E+01	0	0	0	0	0	5.E+02	6.E+02	2.E+03	0	2.E+02	0	0	0	Aromatic compound	
**Benzophenone**	0	0	0	0	0	0	0	0	1.E+01	2.E+04	0	2.E+02	0	3.E+02	0	0	8.E+02	0	0	0	0	0	0	1.E+02	Ketone	
**Benzoyl bromide**	7.E+02	5.E+02	0	3.E+02	0	4.E+02	0	0	0	0	0	7.E+02	0	0	2.E+03	0	0	0	0	0	0	0	0	0	DFG	
**Bicyclo[4.2.0]octa-1,3,5-triene-7,8-dione**	0	1.E+02	0	0	0	0	2.E+02	0	0	0	6.E+02	1.E+03	0	3.E+01	1.E+03	0	6.E+02	0	9.E+02	7.E+02	0	0	0	8.E+00	Ketone	
**cis-6-Nonenol**	0	0	0	0	0	0	8.E+01	0	0	9.E+02	4.E+02	5.E+02	0	0	4.E+02	0	5.E+02	3.E+02	1.E+03	2.E+02	2.E+01	2.E+02	2.E+02	0	Alcohol	*
**Cyclohexaneacetic acid**	0	0	7.E+01	0	0	0	8.E+01	0	0	5.E+02	3.E+02	8.E+02	0	0	4.E+02	0	6.E+02	0	2.E+03	0	0	6.E+01	6.E+01	2.E+01	Carboxylic acid	
**Dec-2-yl 2-fluoroethyl ester glutaric acid**	7.E+01	0	0	0	0	0	0	1.E+02	0	2.E+02	3.E+02	0	0	3.E+02	0	0	2.E+02	0	4.E+02	4.E+02	4.E+02	4.E+02	0	0	DFG	
**Decane**	0	0	0	2.E+02	0	0	2.E+02	5.E+01	2.E+02	1.E+03	7.E+02	8.E+02	0	0	6.E+02	3.E+01	6.E+02	1.E+03	2.E+03	8.E+02	7.E+02	7.E+02	3.E+02	0	Alkane	
**Dibromomethane**	3.E+02	4.E+02	6.E+02	5.E+02	7.E+02	7.E+02	1.E+03	8.E+02	2.E+02	3.E+02	6.E+02	7.E+02	1.E+03	2.E+03	1.E+03	1.E+03	1.E+02	3.E+01	5.E+02	4.E+02	6.E+02	2.E+02	2.E+03	1.E+03	Halogenated hydrocarbon	
**Diethoxymethane**	2.E+02	2.E+02	0	3.E+02	2.E+02	0	8.E+02	2.E+02	0	2.E+03	7.E+02	1.E+03	3.E+01	0	4.E+02	0	1.E+02	1.E+03	8.E+03	5.E+02	0	1.E+03	0	0	Ether	
**Dimethyl sulphide**	1.E+03	1.E+03	2.E+03	9.E+02	1.E+03	1.E+03	0	3.E+02	2.E+03	8.E+02	2.E+03	8.E+02	2.E+03	2.E+03	2.E+03	1.E+03	0	0	0	0	0	0	0	0	Sulphur containing compound	***
**Di-tert-butyl peroxide**	0	0	0	2.E+03	4.E+03	0	2.E+03	0	8.E+03	0	0	6.E+03	1.E+03	9.E+03	0	0	6.E+03	0	1.E+04	2.E+04	0	0	0	0	Ether	
**Docosane**	0	1.E+04	8.E+03	0	0	7.E+03	0	4.E+02	0	0	4.E+03	0	6.E+03	4.E+04	0	0	0	0	6.E+04	0	2.E+03	0	1.E+02	0	Alkane	
**Dodecanal**	0	0	0	0	0	0	2.E+02	0	0	2.E+03	8.E+02	7.E+02	0	0	3.E+02	0	7.E+02	2.E+02	2.E+03	3.E+02	1.E+02	7.E+02	3.E+02	0	Aldehyde	*
**Epistephamiersine**	5.E+01	7.E+01	3.E+01	0	0	0	0	0	0	5.E+01	2.E+02	2.E+02	0	0	0	0	2.E+02	5.E+01	4.E+02	0	1.E+02	0	0	0	DFG	
**Ethylbenzene**	2.E+02	0	2.E+02	0	1.E+02	0	2.E+02	0	3.E+02	0	5.E+02	0	4.E+02	0	6.E+02	0	0	4.E+02	1.E+03	0	5.E+02	0	4.E+02	0	Aromatic compound	
**Heneicosane**	0	2.E+03	0	0	2.E+03	0	4.E+02	0	4.E+03	7.E+03	5.E+03	3.E+03	2.E+03	2.E+03	0	9.E+02	3.E+03	0	8.E+02	0	7.E+03	2.E+03	0	5.E+03	Alkane	
**Heptanal**	0	0	0	0	0	0	0	0	0	3.E+02	9.E+01	9.E+02	0	3.E+01	1.E+02	0	3.E+02	3.E+02	9.E+02	0	0	2.E+02	0	0	Aldehyde	
**Hexanal**	0	0	0	0	0	0	0	0	0	3.E+02	3.E+01	1.E+03	0	0	0	0	7.E+02	7.E+01	1.E+03	0	1.E+02	2.E+02	0	0	Aldehyde	

Table S3 (cont.).

**Homosalate**	3.E+02	2.E+02	2.E+01	0	3.E+02	3.E+02	1.E+02	0	0	0	2.E+01	0	1.E+03	0	0	0	7.E+02	3.E+02	0	0	0	0	4.E+02	0	DFG	
**Isolongifolene**	2.E+03	9.E+01	2.E+03	1.E+02	5.E+02	2.E+02	0	1.E+02	0	9.E+01	3.E+03	5.E+02	2.E+03	7.E+02	0	4.E+02	1.E+02	0	2.E+03	8.E+01	3.E+03	0	0	2.E+02	Alkene	
**Isopropyl dodecanoate/ Isopropyl laurate**	0	0	7.E+02	0	3.E+02	0	1.E+02	7.E+01	0	4.E+03	4.E+02	2.E+03	0	0	5.E+02	0	1.E+03	0	8.E+02	5.E+02	3.E+02	1.E+03	0	3.E+02	Ester	
**Limonene**	5.E+01	0	3.E+01	0	0	0	0	0	1.E+02	0	0	5.E+01	0	0	0	0	1.E+02	4.E+02	2.E+02	0	0	5.E+01	1.E+02	0	Alkene	
**m-Aminophenyl trifluoromethyl ether**	3.E+02	5.E+02	4.E+02	0	0	0	4.E+02	0	0.E+00	9.E+02	0	0	0	1.E+03	9.E+02	0	1.E+03	0	2.E+03	1.E+03	0	1.E+03	1.E+03	1.E+03	DFG	
**Mesitylene**	2.E+02	3.E+02	1.E+02	1.E+02	0	5.E+00	0	0	5.E+02	5.E+02	4.E+02	4.E+02	3.E+02	5.E+02	4.E+02	0	8.E+02	6.E+02	1.E+03	9.E+02	6.E+02	7.E+02	5.E+02	6.E+02	Aromatic compound	
**Methanesulfonyl azide**	3.E+02	0	4.E+03	0	2.E+03	0	0	0	8.E+03	1.E+04	4.E+03	1.E+02	1.E+03	0	2.E+03	0	3.E+03	0	4.E+04	0	0	0	0	0	DFG	
**Methyl 2,3,5-trichloro-4-methoxybenzoate**	0	5.E+01	0	7.E+01	0	2.E+02	0	2.E+02	0	1.E+03	0.E+00	0.E+00	3.E+02	1.E+03	0	2.E+02	0	0	0	0	0	0	0	0	DFG	
**Methyl isohexadecanoate**	0	1.E+02	3.E+01	0	9.E+01	9.E+00	1.E+02	0	0	1.E+03	2.E+02	2.E+03	2.E+01	5.E+01	5.E+02	0	4.E+03	0	2.E+02	0	1.E+02	1.E+02	0	5.E+02	Ester	
**Methyltriglycol acetate**	4.E+01	0	0	0	0	0	3.E+01	2.E+01	0	6.E+01	8.E+01	5.E+01	0	7.E+01	3.E+00	0	4.E+01	6.E+01	1.E+02	1.E+02	1.E+02	0	0	0	DFG	
**N-(4-Methoxybenzyl)-1,3-thiazol-2-amine**	3.E+01	5.E+01	0	0	0	2.E+01	6.E+01	4.E+01	0	2.E+02	0	0	0	0	0	0	1.E+02	8.E+01	0	9.E+01	0	0	0	0	DFG	
**N,3-dimethyl-N-(2-phenylethyl)-benzeneethanamine**	5.E+02	0	1.E+02	0	2.E+02	0	2.E+02	0	2.E+03	0	7.E+02	0.E+00	5.E+02	0	6.E+02	0	0	2.E+03	4.E+03	0	1.E+03	0	4.E+02	0	Nitrogen containing compound	
**N,N-Dimethyl-3-butoxypropylamine**	2.E+01	0	0	0	0	4.E+02	0	2.E+02	0	2.E+03	0	5.E+02	0	1.E+03	1.E+03	0	8.E+02	2.E+03	2.E+03	2.E+02	0	9.E+02	9.E+02	1.E+03	DFG	
**Naphthalene**	5.E+02	0	5.E+02	0	3.E+02	0	3.E+02	0	8.E+02	0	2.E+03	0	9.E+02	0	9.E+02	0	0	2.E+03	3.E+03	0	1.E+03	0	8.E+02	0	Aromatic compound	
**Nonanal**	0	0	0	0	0	0	0	0	0	4.E+03	3.E+03	1.E+04	0	1.E+02	0	0	2.E+03	3.E+03	1.E+04	1.E+03	0	5.E+02	2.E+03	0	Aldehyde	*
**Nonanoic acid**	7.E+02	4.E+02	0	0	0	0	2.E+03	0	0	2.E+03	6.E+03	2.E+04	0	4.E+02	1.E+04	1.E+03	1.E+04	7.E+02	0	2.E+03	4.E+03	5.E+03	0	7.E+03	Carboxylic acid	
**N-phenylethanethioamide**	9.E+01	0	1.E+02	0	9.E+01	0	9.E+01	0	0	0	2.E+02	0	0	0	2.E+02	0	0	0	0	0	0	0	0	0	DFG	
**Octanal**	0	0	0	0	6.E+01	0	0	0	0	9.E+02	5.E+02	4.E+03	0	4.E+00	2.E+02	0	1.E+03	6.E+02	4.E+03	2.E+02	1.E+02	9.E+02	3.E+02	0	Aldehyde	*
**o-cyclopropanecarbonyl-o-'isobutyryl-1,2-benzenediol**	4.E+01	3.E+01	7.E+01	0	8.E+01	0	3.E+01	0	1.E+02	0	0	3.E+01	1.E+02	0	1.E+02	0	2.E+02	1.E+02	4.E+02	2.E+01	2.E+02	1.E+02	0	1.E+01	Ester	
**Phenol**	1.E+02	0	0	4.E+01	0	0	2.E+02	0	0	1.E+03	5.E+02	3.E+02	2.E+02	2.E+02	3.E+02	0	4.E+02	1.E+03	4.E+02	2.E+02	0	0	2.E+01	0	Alcohol	
**Phenyl buta-2,3-dienyl ether**	2.E+01	0	0	0	9.E+00	0	2.E+01	0	9.E+01	0	7.E+01	0	0	0	3.E+01	0	0	2.E+02	2.E+02	0	5.E+01	0	7.E+01	0	Ether	
**p-Toluic acid, tridec-2-ynyl ester**	0	0	3.E+02	0	2.E+02	4.E+02	1.E+02	0	6.E+02	4.E+03	4.E+02	0	0	0	5.E+02	0	0	7.E+01	8.E+02	2.E+02	2.E+03	2.E+03	0	0	DFG	
**p-Xylene**	9.E+02	0	1.E+03	0	5.E+02	0	9.E+02	0	2.E+03	0	2.E+03	0	3.E+03	0	2.E+03	0	0	2.E+03	4.E+03	0	3.E+03	0	2.E+03	0	Aromatic compound	
**Styrene**	3.E+02	2.E+02	3.E+02	1.E+02	2.E+02	2.E+01	1.E+02	7.E+01	9.E+02	5.E+02	9.E+02	5.E+02	5.E+02	3.E+02	7.E+02	0	5.E+02	9.E+02	2.E+03	5.E+02	8.E+02	6.E+02	8.E+02	3.E+02	Aromatic compound	
**Tert-butyl ester salicylic acid**	0	0	3.E+02	0	0	0	0	0	0	1.E+02	8.E+02	6.E+02	0	0	1.E+03	0	1.E+04	0	3.E+01	0	1.E+02	0	0	2.E+03	DFG	
**Tetrachloroethylene**	0	0	0	0	0	0	0	0	0	0	0	0	0	0	0	0	5.E+01	0	0	0	0	4.E+01	5.E+01	7.E+01	Halogenated hydrocarbon	
**Tetradecane**	0	0	1.E+03	0	0	0	0	1.E+02	0	3.E+03	0	4.E+03	0	8.E+02	0	7.E+01	4.E+03	1.E+03	0	1.E+03	0	2.E+03	0	9.E+02	Alkane	
**Toluene**	1.E+03	0	1.E+03	0	6.E+02	0	8.E+02	0	3.E+03	0	3.E+03	0	4.E+03	0	2.E+03	0	0	3.E+03	7.E+03	0	5.E+03	0	1.E+03	0	Aromatic compound	
**trans-1,4-Cyclohexanediol, bis(heptafluorobutyrate)**	0	0	0	0	0	2.E+02	0	0	0	0	0	1.E+02	2.E+02	0	2.E+02	1.E+02	0	0	0	0	0	3.E+02	2.E+02	3.E+02	DFG	
**UC(14.243)**	0	2.E+02	0	7.E+01	0	8.E+01	0	5.E+01	0	2.E+02	0	2.E+02	0	2.E+02	0	5.E+01	7.E+02	0	0	3.E+02	0	3.E+02	0	1.E+02	Unclassified	
**UC(14.82)**	1.E+02	9.E+01	4.E+01	9.E+01	0	2.E+01	2.E+02	2.E+01	0	5.E+02	2.E+02	2.E+02	2.E+02	2.E+02	3.E+01	0	6.E+01	4.E+02	6.E+02	1.E+02	4.E+02	2.E+02	0	2.E+01	Unclassified	
**UC(19.747)**	5.E+01	1.E+02	5.E+01	0	0	0	8.E+01	6.E+01	1.E+02	1.E+02	2.E+02	1.E+02	4.E+02	5.E+02	0	0	3.E+02	1.E+02	9.E+02	5.E+02	4.E+02	3.E+02	5.E+01	0	Unclassified	
**UC(27.28)**	5.E+01	1.E+02	6.E+01	5.E+01	0	3.E+01	2.E+00	4.E+00	2.E+02	2.E+02	1.E+02	3.E+02	2.E+02	2.E+02	1.E+02	0	3.E+02	0.E+00	5.E+02	4.E+02	3.E+02	0	2.E+02	3.E+02	Unclassified	
**UC(27.756)**	5.E+01	2.E+02	0	0	2.E+02	1.E+02	3.E+01	4.E+01	0	5.E+02	3.E+02	7.E+02	6.E+01	4.E+01	4.E+02	4.E+01	5.E+02	0	0	8.E-01	0	0	4.E+01	0	Unclassified	
**UC(33.81)**	1.E+02	0	0	0	4.E+02	9.E+01	3.E+02	1.E+02	0	1.E+03	0	0	2.E+02	8.E+02	1.E+02	1.E+02	0	0	0	0	0	0	1.E+02	0	Unclassified	
**UC(35.86)**	0	6.E+01	0	0	0	0	3.E+02	0	0	3.E+02	8.E+02	3.E+03	0	2.E+02	2.E+03	0	2.E+03	6.E+01	0	0	0	5.E+02	0	7.E+02	Unclassified	
**UC(36.84)**	5.E+02	0	4.E+02	0	2.E+02	0	2.E+02	0	1.E+03	0	1.E+03	0	8.E+02	0	3.E+02	0	0	2.E+03	3.E+03	0	1.E+03	0	6.E+02	0	Unclassified	
**UC(37.94)**	5.E+01	0	0	0	0	0	3.E+01	2.E+01	0	2.E+02	8.E+01	7.E+01	0	8.E+01	1.E+02	0	4.E+01	0	5.E+02	2.E+02	0	9.E+01	1.E+02	6.E+01	Unclassified	
**UC(39.23)**	0	0	0	0	0	0	0	0	0	9.E+02	4.E+02	1.E+02	0	2.E+02	3.E+02	0	2.E+02	0	6.E+02	0	0	0	3.E+02	0	Unclassified	
**UC(41.47)**	7.E+01	0	6.E+01	0	0	0	0	0	0	0	2.E+02	0	1.E+02	0	1.E+02	0	0	3.E+02	4.E+02	0	2.E+02	0	1.E+02	0	Unclassified	
**UC(41.51)**	0	0	0	0	0	0	8.E+01	0	0	0	2.E+02	3.E+02	0	0	1.E+02	0	2.E+02	0	4.E+02	0	2.E+02	0	2.E+02	0	Unclassified	
**UC(42.08)**	0	0	6.E+01	0	0	0	0	0	4.E+02	0	9.E+02	0	3.E+02	0	2.E+01	0	0	9.E+02	1.E+03	0	2.E+02	0	5.E+00	0	Unclassified	
**UC(42.13)**	9.E+01	0	0	0	0	0	9.E+01	0	2.E+02	0	3.E+02	0	3.E+02	0	2.E+02	0	0	0	5.E+02	0	0	0	2.E+02	0	Unclassified	
**UC(42.15)**	0	3.E+01	2.E+02	0	2.E+02	0	2.E+01	0	4.E+01	1.E+03	0	1.E+01	0	7.E+01	9.E+00	0	9.E+01	0	5.E+02	1.E+01	0	0	3.E+01	0	Unclassified	
**UC(42.61)**	2.E+02	0	1.E+02	0	0	0	9.E+01	0	0	0	4.E+02	0	3.E+02	0	2.E+02	0	0	6.E+02	7.E+02	0	3.E+02	0	2.E+02	0	Unclassified	

Table S3 (cont.).

**UC(43.02)**	0	6.E+01	0	7.E+01	0	5.E+01	0	7.E+01	0	1.E+02	0	1.E+02	0	2.E+02	0	4.E+01	2.E+02	9.E+01	0	3.E+02	0	1.E+02	0	3.E+02	Unclassified	
**UC(43.09)**	0	0	0	0	0	1.E+02	0	0	0	4.E+02	0	2.E+02	0	2.E+02	0	0	3.E+02	0	0	3.E+02	0	3.E+02	0	3.E+02	Unclassified	
**UC(43.62)**	9.E+01	9.E+01	0	5.E+01	0	6.E+01	9.E+01	5.E+01	0	0	3.E+02	2.E+02	0	2.E+02	2.E+02	1.E+02	3.E+02	0	0	3.E+02	3.E+02	0	2.E+02	2.E+02	Unclassified	
**UC(44.31)**	0	0	0	0	7.E+00	3.E+01	2.E+01	9.E-01	0	0	0	0	0	3.E+01	0	3.E+00	0	0	8.E+01	6.E+00	0	0	1.E+00	0	Unclassified	
**UC(44.35)**	6.E-01	1.E+02	1.E+02	0	8.E+01	0	4.E+01	0	2.E+02	6.E+02	4.E+01	0	2.E+02	0	2.E+02	0	3.E+03	1.E+03	3.E+02	1.E+02	3.E+02	5.E+01	4.E+02	0	Unclassified	
**UC(44.37)**	1.E+00	4.E+01	7.E+01	0	0	0	4.E+01	2.E+01	0	2.E+02	2.E+02	3.E+02	0	1.E+02	3.E+02	0	5.E+02	4.E+01	4.E+02	6.E+00	2.E+02	2.E+02	0	0	Unclassified	
**UC(44.45)**	0	0	3.E+01	0	9.E+01	0	4.E+01	0	0	2.E+02	0	4.E+01	6.E+01	2.E+00	1.E+02	0	0	0	0	8.E+00	9.E+01	0	0	3.E-01	Unclassified	
**UC(44.59)**	0	7.E+01	0	0	2.E+02	0	9.E+02	0	1.E+02	4.E+02	5.E+02	0	0	5.E+02	0	0	0	3.E+02	7.E+02	3.E+02	4.E+02	7.E+02	0	0	Unclassified	
**UC(45.46)**	1.E+02	2.E+02	0	0	0	0	2.E+02	0	0	5.E+02	0	4.E+02	0	0	3.E+02	0	5.E+02	3.E+02	7.E+02	4.E+02	0	6.E+02	4.E+02	0	Unclassified	
**UC(45.55)**	9.E+01	8.E+01	0	0	2.E+01	0	3.E+01	0	0	0	0	2.E+02	0	0	0	0	0	2.E+02	0	0	1.E+02	0	0	0	Unclassified	
**UC(45.60)**	4.E+01	2.E+01	0	0	3.E+01	0	0	0	1.E+02	0	2.E+02	0	1.E+02	0	2.E+02	0	0	2.E+02	3.E+02	2.E+02	1.E+02	0	1.E+02	0	Unclassified	
**UC(45.67)**	0	0	2.E+02	0	1.E+02	0	9.E+00	0	0	2.E+03	1.E+02	5.E+02	0	9.E+00	9.E+01	0	9.E+02	0	4.E+02	1.E+02	0	2.E+02	0	0	Unclassified	
**UC(46.40)**	1.E+01	0	1.E+02	0	4.E+01	2.E+01	8.E+01	0	0	5.E+02	4.E+02	1.E+02	2.E+01	0	1.E+02	0	0	0	3.E+02	0	2.E+02	2.E+02	0	0	Unclassified	
**UC(47.66)**	0	1.E+02	0	4.E+01	0	0	0	3.E+01	3.E+03	4.E+03	0	0	0	2.E+02	0	9.E+01	0	9.E+02	2.E+02	4.E+02	0	0	0	0	Unclassified	
**Z-4-Dodecenol**	0	0	0	0	0	0	2.E+02	0	0	2.E+03	1.E+03	2.E+03	0	0	1.E+03	0	1.E+03	5.E+02	2.E+03	5.E+02	0	5.E+02	8.E+02	0	Alcohol	

Table S4. Bacterial taxa (ASV counts) detected throughout dataset. All bacteria that were detected in aposymbiotic anemones, anemones symbiotic with *B. minutum symbiosis* (homologous symbiosis) and anemones symbiotic with *D. trenchii* (heterologous symbiosis). Bacteria had to be detected in at least three replicates in at least one symbiotic state to be included. Significance was determined using differential abundance testing and the number of asterisks denotes the size of the adjusted p-value: *<0.05, **<0.01, ***<0.001.

Name	Taxon ID	Aposymbiotic anemone					Heterologous symbiosis					Homologous symbiosis					Significance
Aestuariibacter halophilus	taxon_60	98	0	256	58	0	37	115	90	103	48	103	0	117	32	0	
Alphaproteobacteria	taxon_15	3744	929	1706	4441	1243	0	49	706	0	11	0	18	0	0	0	
Alphaproteobacteria	taxon_106	146	0	7	26	84	0	0	0	0	0	0	0	0	0	12	*
Alphaproteobacteria	taxon_111	95	0	2	58	0	13	30	8	0	43	0	0	0	0	0	*
Alphaproteobacteria	taxon_113	62	0	17	162	0	0	0	0	0	0	0	0	0	0	0	*
Alphaproteobacteria	taxon_115	0	5	9	99	23	4	3	27	20	3	0	18	16	0	0	
Alphaproteobacteria	taxon_140	13	0	0	14	0	0	3	57	0	48	0	0	0	0	0	
Alphaproteobacteria	taxon_173	12	0	0	22	18	0	0	21	0	0	0	7	0	0	0	
Alphaproteobacteria	taxon_184	34	0	12	17	0	0	0	0	0	0	0	0	0	0	0	
Alphaproteobacteria	taxon_194	19	4	0	0	4	2	0	0	15	0	0	0	15	0	0	
Alphaproteobacteria	taxon_261	0	0	0	0	0	0	7	5	14	0	4	0	0	0	0	
Alphaproteobacteria	taxon_318	6	0	4	10	0	0	0	0	0	0	0	0	0	0	0	
Alteromonadaceae	taxon_64	153	17	67	141	19	61	111	143	104	56	49	0	20	25	0	
Alteromonadaceae	taxon_95	0	0	0	0	14	0	0	0	0	0	103	206	13	40	0	**
Alteromonadaceae	taxon_131	0	0	0	0	0	0	0	0	0	0	31	22	37	0	72	***
Alteromonas sp.	taxon_49	229	36	121	344	39	52	68	65	151	169	244	131	96	77	43	
Alteromonas sp.	taxon_71	110	0	84	110	36	0	45	0	27	36	107	0	70	47	0	
Alteromonas simiduii	taxon_85	64	0	61	112	0	0	40	23	21	54	56	16	26	0	8	
Arcobacter sp.	taxon_18	5479	150	2619	9	0	0	0	0	0	0	0	0	0	0	0	**
Bacteroidetes	taxon_8	17054	0	1466	1640	853	0	0	0	0	0	0	0	0	0	0	***
Bacteroidetes	taxon_29	3623	0	303	287	254	0	0	0	0	0	0	0	0	0	0	***
Bacteroidetes	taxon_35	0	0	0	0	0	29	0	0	0	0	25	1054	1113	516	186	***
Bacteroidetes	taxon_54	93	36	503	525	53	3	35	0	11	0	23	12	26	0	9	*
Bacteroidetes	taxon_235	29	0	3	5	0	0	0	0	0	0	0	0	0	0	0	
Bacteroidetes	taxon_247	12	0	0	14	8	0	0	0	0	0	0	0	0	0	0	
Balneola alkaliphila	taxon_262	5	0	16	8	0	0	0	0	0	0	0	0	0	0	0	
Betaproteobacteria	taxon_170	27	0	9	47	0	0	0	0	0	0	0	0	0	0	0	
Campylobacteraceae	taxon_2	6535	5882	1892	20835	5691	0	0	0	0	0	0	0	0	0	0	***
Chlamydiia sp.	taxon_79	0	0	0	0	0	0	0	0	0	0	25	359	0	0	149	**
Chlamydiia sp.	taxon_150	0	26	61	14	0	0	0	0	0	0	0	0	0	11	0	
Chlamydiia sp.	taxon_176	0	0	0	0	0	0	0	0	0	0	0	27	45	0	3	**
Cohaesibacter sp.	taxon_50	1398	19	68	300	58	0	0	0	0	0	0	0	0	0	10	***
Croceibacter atlanticus	taxon_68	603	7	86	127	9	0	0	0	0	0	3	0	7	0	0	***
Crocinitomicaceae	taxon_1	6255	21	2012	17302	777	341	2582	2088	6778	5158	395	156	266	221	346	
Cutibacterium acnes	taxon_167	13	2	0	0	0	0	0	42	0	11	5	0	0	4	5	
Cytophagales	taxon_121	0	0	0	0	0	0	3	0	186	24	0	0	0	0	0	*
Devosia subaequoris	taxon_236	0	2	11	0	18	0	0	0	0	6	0	0	0	0	0	
Donghicola eburneus	taxon_57	276	39	65	236	22	0	18	41	318	171	0	97	0	0	6	*
Ekhidna lutea	taxon_179	0	0	0	21	0	4	9	0	0	0	9	17	0	0	10	
Erythrobacter gaetbuli	taxon_175	0	0	0	0	0	5	10	16	0	45	0	0	0	0	0	
Flavobacteriaceae	taxon_4	16491	299	5693	2526	0	22	29	127	305	402	0	0	161	0	0	*
Flavobacteriaceae	taxon_22	3729	0	322	365	135	24	147	258	445	767	15	682	114	0	198	
Flavobacteriaceae	taxon_36	168	20	97	15	0	0	0	578	0	432	350	1091	0	146	21	
Flavobacteriaceae	taxon_69	279	5	125	129	22	11	38	10	7	19	9	0	102	26	15	
Flavobacteriaceae	taxon_76	513	0	46	6	0	0	0	0	0	0	0	0	0	0	0	*
Flavobacteriaceae	taxon_78	0	0	6	0	0	0	3	62	180	0	0	277	30	0	0	
Flavobacteriaceae	taxon_87	339	0	39	38	10	0	9	0	0	16	0	0	0	5	7	
Flavobacteriaceae	taxon_122	102	10	53	18	0	0	0	0	0	0	0	14	0	0	0	**
Flavobacteriaceae	taxon_135	33	0	42	21	0	0	0	24	0	8	0	0	25	0	0	
Flavobacteriaceae	taxon_165	36	6	0	0	33	0	0	0	0	14	0	0	0	0	0	
Flavobacteriales	taxon_74	31	10	180	49	86	8	46	0	8	0	7	0	150	0	17	
Flavobacteriales	taxon_146	0	0	0	0	0	0	4	0	0	0	7	61	52	0	0	*
Flavobacteriia	taxon_30	3276	31	878	199	17	0	0	0	0	6	0	0	0	0	0	**
Gammaproteobacteria	taxon_9	2120	596	1707	3438	2339	460	990	1365	203	719	735	2172	1209	46	156	

Table S4 (cont.).

Gammaproteobacteria	taxon_21	1035	358	998	1078	1094	90	0	546	332	124	231	1133	369	35	125	
Gammaproteobacteria	taxon_24	1510	1180	98	690	3452	0	0	0	0	0	0	0	0	0	0	***
Gammaproteobacteria	taxon_25	691	303	826	870	736	40	17	462	337	103	176	860	313	39	146	
Gammaproteobacteria	taxon_26	234	173	1130	652	463	101	8	38	2028	248	237	304	74	112	17	
Gammaproteobacteria	taxon_47	180	44	150	52	98	12	43	766	194	179	38	180	26	48	0	
Gammaproteobacteria	taxon_61	255	31	419	158	86	0	0	0	0	0	0	11	62	0	15	***
Gammaproteobacteria	taxon_102	11	0	0	0	35	0	10	21	12	45	17	136	19	0	0	
Gammaproteobacteria	taxon_126	0	20	0	46	117	0	0	0	0	0	0	0	0	0	0	*
Gammaproteobacteria	taxon_128	0	0	0	0	0	0	23	5	126	29	0	0	0	0	0	***
Gammaproteobacteria	taxon_143	0	0	0	0	0	0	0	0	8	0	6	111	9	0	0	*
Gammaproteobacteria	taxon_174	0	0	0	0	5	6	0	29	31	8	0	0	0	0	0	
Hyphomonadaceae	taxon_109	76	0	43	4	16	15	11	12	22	12	0	0	9	34	4	
Hyphomonas sp.	taxon_103	42	11	26	14	77	0	19	64	8	33	0	0	0	8	3	*
Labrenzia sp.	taxon_161	0	0	0	0	0	0	0	0	0	0	0	80	11	0	4	**
Leeuwenhoekiella aequorea	taxon_53	377	46	229	194	94	32	11	62	26	48	43	87	61	98	0	
Limimaricola sp.	taxon_84	32	0	4	35	4	34	10	128	57	42	7	14	97	0	19	
Limimaricola cinnabarinus	taxon_240	0	0	0	0	0	12	0	14	5	0	5	0	0	0	0	
Maricaulis maris	taxon_46	411	60	280	144	201	24	133	185	220	133	17	41	79	91	4	
Marinilabiliales	taxon_99	204	0	0	18	130	0	0	0	0	0	0	0	0	0	0	*
Marinobacter salarius	taxon_89	74	8	110	65	36	9	0	29	39	29	9	24	9	13	5	
Marinoscillum sp.	taxon_198	0	0	14	15	0	0	4	0	12	8	0	0	0	5	0	
Maritalea sp.	taxon_107	0	0	23	48	57	0	0	0	0	20	17	37	67	0	0	
Maritalea porphyrae	taxon_56	292	30	49	157	245	41	40	32	10	0	119	156	89	25	11	
Maritalea porphyrae	taxon_75	106	161	27	80	27	0	21	101	0	50	0	0	0	0	0	***
Myxococcales	taxon_66	31	0	0	0	0	0	30	64	56	59	0	617	0	0	0	*
Oceanospirillaceae	taxon_51	488	0	339	0	0	0	0	364	0	0	51	0	330	56	8	
Oceanospirillaceae	taxon_120	87	24	0	25	80	0	0	0	0	0	0	0	0	0	0	**
Oceanospirillales	taxon_130	0	6	41	12	91	0	3	0	0	0	0	0	19	0	0	
Oligoflexia	taxon_230	9	0	0	23	6	0	0	0	0	0	0	0	0	0	0	
Owenweeksia sp.	taxon_27	1702	105	1655	284	157	38	36	95	182	137	17	471	117	21	7	
Paraglaciecola sp.	taxon_63	0	0	0	9	0	0	0	20	0	0	27	267	611	29	7	***
Parasphingorhabdus sp.	taxon_169	0	0	0	0	0	0	0	0	0	9	0	61	10	5	0	*
Photobacterium gaetbulicola	taxon_40	202	100	693	340	138	0	70	317	167	55	116	406	80	79	0	
Photobacterium gaetbulicola	taxon_52	217	42	337	214	89	0	0	156	53	0	48	240	34	0	6	*
Photobacterium gaetbulicola	taxon_88	35	0	135	64	29	0	0	100	27	0	0	73	0	0	0	
Phyllobacteriaceae	taxon_77	193	27	78	79	0	0	0	0	37	91	0	0	39	18	0	
Pleionea mediterranea	taxon_136	44	3	4	0	20	3	20	34	8	5	0	0	9	0	0	
Polaribacter huanghezhanensis	taxon_62	778	8	106	125	17	0	0	0	0	0	0	0	0	0	0	***
Proteobacteria	taxon_94	87	26	69	0	80	28	11	24	0	55	0	0	0	0	0	*
Proteobacteria	taxon_114	0	0	26	158	0	0	0	8	17	0	0	0	12	13	4	
Proteobacteria	taxon_152	13	0	14	13	31	0	0	37	0	0	0	0	0	0	0	
Proteobacteria	taxon_155	0	0	0	0	0	0	0	0	0	0	7	52	26	6	14	***
Proteobacteria	taxon_166	0	0	0	0	0	5	5	16	12	15	0	36	0	0	0	
Pseudoalteromonas sp.	taxon_92	66	16	65	47	26	0	9	22	46	0	0	48	34	20	0	
Pseudoalteromonas arabiensis	taxon_37	595	114	491	293	244	17	21	240	196	88	107	283	133	48	16	
Pseudoalteromonas piscicida	taxon_93	180	0	90	96	19	0	0	0	0	0	0	0	0	0	0	***
Pseudoteredinibacter isoporae	taxon_112	112	0	6	108	0	0	0	0	0	0	10	0	7	0	0	
Rhizobiales	taxon_65	0	0	0	0	9	3	5	6	398	409	0	101	0	0	0	**
Rhizobiales	taxon_96	104	0	16	18	29	0	0	61	0	58	6	77	0	4	0	
Rhizobiales	taxon_202	0	0	27	17	10	0	0	0	0	0	0	0	0	0	0	
Rhizobium subbaraonis	taxon_41	962	70	297	193	95	50	66	147	39	291	51	80	140	35	69	
Rhodobacteraceae	taxon_17	2540	285	503	2494	1668	36	494	518	1232	512	0	66	106	66	0	*
Rhodobacteraceae	taxon_23	850	68	355	646	84	170	220	1221	534	981	194	875	755	64	50	
Rhodobacteraceae	taxon_45	905	9	0	138	47	33	63	72	429	401	0	0	0	0	0	***
Rhodobacteraceae	taxon_104	231	0	0	16	48	0	0	0	0	0	0	0	0	0	0	*
Rhodobacteraceae	taxon_117	0	0	0	0	0	0	0	0	0	0	0	148	8	63	3	***
Rhodobacteraceae	taxon_118	99	5	28	0	55	0	0	0	6	18	0	0	10	0	0	
Rhodobacteraceae	taxon_129	42	0	0	12	42	0	0	35	0	0	9	34	0	0	8	
Rhodobacteraceae	taxon_133	12	0	0	121	24	0	0	0	0	0	0	0	0	0	0	*

Table S4 (cont.).

Rhodobacteraceae	taxon_145	24	0	11	37	0	0	16	31	8	0	0	0	0	0	0	
Rhodobacteraceae	taxon_157	46	0	0	19	0	0	0	0	0	0	0	14	15	0	6	
Rhodobacteraceae	taxon_241	0	0	0	0	0	0	5	27	0	4	0	0	0	0	0	
Rhodospirillales	taxon_187	0	0	0	0	0	0	0	0	0	0	0	39	13	0	11	
Unclassified	taxon_38	0	13	0	0	0	0	0	0	0	248	5	2086	0	11	517	
Unclassified	taxon_67	173	98	61	100	412	2	0	0	0	0	0	0	0	0	0	***
Unclassified	taxon_72	155	0	0	3	0	0	20	0	229	164	0	94	0	0	0	
Unclassified	taxon_73	317	9	7	68	0	20	8	23	0	61	6	27	79	0	20	
Unclassified	taxon_83	0	21	107	113	102	43	0	40	13	0	0	50	8	3	0	
Unclassified	taxon_86	433	0	17	15	0	0	0	0	0	0	0	0	0	0	0	*
Unclassified	taxon_119	0	0	0	0	0	0	68	0	3	147	0	0	0	0	0	*
Unclassified	taxon_123	43	0	0	15	0	5	2	0	66	64	0	0	0	0	0	
Unclassified	taxon_144	34	0	33	22	0	7	0	16	0	10	0	0	6	0	0	
Unclassified	taxon_149	20	0	0	0	58	3	0	0	12	20	0	0	0	0	0	
Unclassified	taxon_172	0	8	6	0	20	6	8	0	0	16	0	0	17	0	0	
Staphylococcus sp.	taxon_227	10	0	6	23	0	0	0	0	0	0	0	0	0	0	0	
Sulfitobacter sp.	taxon_116	41	9	6	25	0	0	7	18	47	22	0	35	0	13	0	
Tepidibacter mesophilus	taxon_33	553	168	401	1403	638	0	0	0	0	13	0	0	0	0	0	***
Thalassospira sp.	taxon_82	66	13	36	159	0	0	11	52	69	0	18	11	60	12	0	
Thalassospira sp.	taxon_141	12	0	10	15	29	0	0	0	0	0	0	50	19	0	0	
Thalassospira xiamenensis	taxon_178	23	0	18	30	0	0	0	0	0	0	0	0	0	0	0	
Thalassotalea sp.	taxon_34	652	58	356	285	313	102	102	274	338	141	112	241	71	54	0	
Thalassotalea ganghwensis	taxon_10	3031	457	1862	1556	1710	611	545	1718	1763	744	562	1188	529	492	0	
Vibrio sp.	taxon_13	4753	536	872	1837	1637	130	45	105	1478	659	121	654	125	48	0	
Vibrio crosai	taxon_11	4427	3790	1512	3078	2361	48	16	73	77	156	46	400	477	0	28	
Vibrio crosai	taxon_39	813	636	268	517	367	0	0	61	24	0	0	82	53	0	0	**
Vibrio crosai	taxon_42	769	547	293	445	404	0	0	0	0	0	0	64	49	0	0	***
Vibrio sinaloensis	taxon_43	626	579	343	404	382	0	0	22	0	0	0	38	29	0	0	***
Vibrio sinaloensis	taxon_44	688	556	235	399	328	0	0	33	0	0	0	45	106	14	0	***
Vibrionaceae	taxon_19	3247	374	599	1534	1232	48	0	100	759	50	0	131	12	0	0	**
Vibrionaceae	taxon_20	3395	431	565	1440	1078	0	29	98	725	75	0	0	0	0	0	***
Vibrionaceae	taxon_55	274	63	306	490	162	0	0	0	0	0	0	0	10	0	0	***
Vibrionaceae	taxon_70	198	24	22	89	84	8	0	9	106	68	19	78	0	0	0	*
Zhongshania sp.	taxon_200	14	5	4	0	0	0	0	16	0	16	0	0	0	0	0	
